# Predicting Healthy Lifestyle Behaviours Among Patients With Type 2 Diabetes in Rural Bali, Indonesia

**DOI:** 10.1177/1179551420915856

**Published:** 2020-04-20

**Authors:** Nice Maylani Asril, Keiji Tabuchi, Miwako Tsunematsu, Toshio Kobayashi, Masayuki Kakehashi

**Affiliations:** 1 Department of Health Informatics, Graduate School of Biomedical and Health Science, Hiroshima University, Hiroshima, Japan; 2 Faculty of Education, Ganesha University of Education, Bali, Indonesia; 3 Department of Health Promotion and Development Science, Graduate School of Biomedical and Health Science, Hiroshima University, Hiroshima, Japan; 4 Department of General Internal Medicine, Ishii Memorial Hospital, Yamaguchi, Japan

**Keywords:** Diabetes, demographic characteristics, clinical and lifestyle factors, diabetes knowledge, extended health belief model, healthy lifestyle behaviour, diabetes management, Indonesia

## Abstract

**Background:**

Type 2 diabetes is a lifelong metabolic disease closely related to unhealthy lifestyle behaviours. This study aimed to identify factors explaining the healthy lifestyle behaviours of patients with type 2 diabetes in rural Indonesia. The extended health belief model, demographic characteristics, clinical lifestyle factors and diabetes knowledge were investigated to provide a complete description of these behaviours.

**Method:**

A sample of 203 patients with type 2 diabetes representing a cross-section of the population were recruited from community health centres in the rural areas of Bali province. The data were collected through questionnaires. Descriptive statistics and a hierarchical regression test were employed.

**Results:**

This study showed demographic characteristics, clinical and lifestyle factors, diabetes knowledge and the extended health belief model accounted for 71.8% of the variance in healthy lifestyle behaviours of patients with type 2 diabetes in rural Indonesia. The significant demographic factors were age, education level, employment status and traditional beliefs. The significant clinical and lifestyle factors were alcohol use, diabetic medicine and duration of symptoms. Finally, the significant extended health belief model factors were perceived severity, susceptibility, barriers, family support, bonding social capital and chance locus of control.

**Conclusions:**

The extended health belief model forms an adequate model for predicting healthy lifestyle behaviours among patients with diabetes in rural Indonesia. The contribution of this model should be strengthened in developing the diabetes management.

## Introduction

Diabetes mellitus is a global health problem that is very important to note because of the high prevalence, complications and mortality and because of the enormous health care costs.^1-3^ World Health Organization (WHO) in 2019 classified diabetes mellitus based on clinical care into (a) type 1 diabetes, caused by absolute insulin deficiency, this type of diabetes is found in children and adults; (b) type 2 diabetes, the causes of this disease vary from insulin resistance and insulin deficiency to insulin hormone imbalance; (c) hybrid forms of diabetes, including slowly evolving immune-mediated diabetes and ketosis-prone type diabetes; (d) other specific type of diabetes; (e) unclassified diabetes; and (f) hyperglycaemia first detecting during pregnancy.^4^

Type 2 diabetes has a high morbidity and becomes a major cause of blindness, kidney failure, heart attacks, stroke and lower limb amputation. Data from WHO showed that around 1.6 million deaths before the age of 70 years were directly caused by type 2 diabetes in 2016.^1^ Data-related diabetes complication showed 50% of diabetic patients die because of heart disease. In addition, 2% of the population of diabetic patients become blind, 10% suffer from serious vision problems, 10% to 20% die from kidney failure and 50% experience nerve damage (neuropathic diabetes).^1^

Type 2 diabetes rates rose significantly in many middle-income to low-income countries, including Indonesia.^1,2^ In 2016, type 2 diabetes prevalence in Indonesia was about 18 million people (7%) with risk factors such as overweight, obesity and physical inactivity.^3^ These risk factors were closely related to unhealthy lifestyles. Practicing a healthy lifestyle is the primary means of reducing the risk factors of diabetes complications.^4^

Surveys showed a fivefold rise in the prevalence of type 2 diabetes in the rural areas of developing countries from 1985 to 2010.^5^ The rate increased in Thailand and Korea as communities urbanized.^6^ Bali is one of the provinces in Indonesia starting to experience a higher burden of noncommunicable diseases.^7^ The increasing prevalence of type 2 diabetes is also evident in rural Bali, Indonesia. The people in this area are facing several obstacles in finding treatments for their diseases. Some of these obstacles are a high level of poverty, limited access to insurance and health service delivery. As a consequence, a high percentage of the population treat themselves or use traditional medicine from shaman.^8^ Type 2 diabetes is one of the top 10 diseases managed by Buleleng health care facilities.^7^ Buleleng, consisting mostly of rural areas, has the second highest type 2 diabetes prevalence of regencies in Bali.^9^

In 2016, type 2 diabetes and its complications accounted for nearly 6% of deaths in Indonesia.^3^ This situation was exacerbated by the lack of resources for screening, diagnosing and treating diabetes in Indonesia’s primary care facilities.^10^ The basic method for diagnosing diabetes in primary care facilities is by measuring blood glucose content.^3^ Another critical issue is that the number of qualified diabetic educators is limited and most of them live in large cities.^11^

Few studies on type 2 diabetes have been conducted in rural Indonesia because of obstacles such as geography, lack of interest and tools for diagnosis.^12^ Hence, more research is required to reduce the fear of diabetes in rural communities. Furthermore, type 2 diabetes is discussed in this study because of those several important issues and the complexity of diabetes management, especially in rural Indonesia.

The extended health belief model (EHBM) describes interventions to encourage healthy lifestyle practices by patients with type 2 diabetes.^13,14^ The EHBM was developed to explain the difficulties faced by patients in recognizing health risks and applying recommended healthy lifestyles.^15^ The EHBM was selected for this study because it reflects a comprehensive examination of factors, including patient attitudes, beliefs and behaviour.^16^ Furthermore, the EHBM has been proved to be adequate to explain sociopsychological factors in diabetic patients.^13,14^ It also includes locus of control (LoC) as one of the social learning theory variables^17^ and social support theory variables.^18^ Unlike the health belief model (HBM) alone which was unable to explain the specific psychological factors in rural Africa and Thailand, EHBM was suitable for analysing health conditions in rural areas.^19,20^ Various components of EHBM have been widely used in study projects and interventions designed for diabetics,^13,14^ but the efficacy of EHBM has not been tested with populations in rural areas.

Demographic characteristics, clinical and lifestyle factors, and diabetes knowledge were also investigated in this study because they have implications for a person’s decision to manage his or her disease.^19,21^ The longitudinal research^22^ showed that demographic characteristics and clinical factors, that is, age between 55 and 65 years, people with obesity (body mass index [BMI] around 30 kg/m^2^ or greater) and high level of fasting blood glucose, were significant predictors of 20 years of incident type 2 diabetes in China. Combined demographic and lifestyle factors, that is, age, sex, race, educational level, annual income, alcohol use and tobacco use, were the strong predictors for prevalence in patients with type 2 diabetes.^23,24^ These factors also influence health opportunities, healthy lifestyle behaviours, onset and outcomes in patients.^1^ Furthermore, diabetes knowledge is also important for the patients to evaluate the quality of their current lifestyle behaviour.^25^

Therefore, the purpose of this study was to determine whether demographic characteristics, clinical and lifestyle factors, diabetes knowledge and the EHBM could be used as a framework for investigating the predictors of healthy lifestyle behaviours of patients with type 2 diabetes in rural areas. This study was expected to provide some information on variables that can be used in developing integrated diabetes management for patients.

## Methods

### Study design

The cross-sectional study was conducted in the local community health centre (*Puskesmas*) in Buleleng between August and October 2017. *Puskesmas* is a community health clinic supervised by the Indonesian Ministry of Health located throughout Indonesia. *Puskesmas* in Buleleng was chosen because it has the highest prevalence of type 2 diabetes^9^ and served people from some rural areas.

The participants were adults with type 2 diabetes detected by blood tests. The symptoms of type 2 patients include increased frequency of urine (polyuria), thirst (polydipsia) and starvation (polyphagia) with unexplained weight loss. The experience of numbness in the extremities, pain in the legs (dysesthesia) and blurred vision are also included in addition to having severe infection. Some patients may present the loss of consciousness or coma but this is less common than in type 1 diabetes.^4^

Only those who could speak and read the Indonesian language were selected for the sample. The exclusion criteria were pregnancy, cognitive impairment, severe or unstable medical conditions (during the past 12 months), severe hypoglycaemia, diabetic ketoacidosis and refusal to give informed consent. The minimum required sample size was 187 estimated using a priori sample size formula for hierarchical regression analysis by Soper^26^ to achieve effect size 0.15, probability level .05 and power 0.80 with 30 predictors.^27^ There were 256 participants recruited, of whom 203 completed and returned the questionnaires.

### Recruitment

The participants were selected voluntarily by convenience sampling through invitations from the head of *Puskesmas* to participate in the study. Participants were divided into 5 groups based on the numbers listed in the invitation. Each group had a separate meeting in which the researchers explained written informed consent to the participants. Informed consent was obtained from all participants in the study. Each participant took about 30 minutes to complete the questionnaires anonymously.

### Measurements

Demographic characteristics, clinical and lifestyle factors, diabetes knowledge, EHBM and healthy lifestyle behaviours were measured using a personal information sheet and standard self-report scales. The scales were translated into Indonesian by the researcher. The back-translation technique was used to achieve a valid Indonesian translation, which was compared with the original English version for discrepancies.^28^ The translated version was submitted to an expert panel to evaluate the validity of its content. The panel consisted of 3 investigators including a medical doctor, a nurse and a psychologist with research expertise in diabetes and a sworn translator. The investigators approved the content validity of all scales in this study through qualitative methods; all the translation of items in scales was improved until saturation was achieved. The reliability of the scales was assessed using Cronbach’s alpha. This study considered that Cronbach’s alpha of >0.7 is adequate.^29^

#### Demographic characteristics

Information on demographic characteristics included age, sex, marital status, education level, religion and employment status collected using a standardized case record form. The traditional beliefs were also measured in this study because Balinese people have the belief that shamans can cure diseases.^30^ The traditional beliefs included the belief that black magic is a cause of diabetes and the belief that shamans can cure the disease.

#### Clinical and lifestyle factors

The BMI was collected using Centers for Disease Control and Prevention charts for adults. Fasting blood glucose level, duration of diabetes symptoms, other diseases, diabetic medicine and family history of diabetes were collected from the medical records. The self-reported tobacco and alcohol use were classified as current users of tobacco and alcohol products (as Yes) and as nonusers of tobacco and alcohol products (as No).

#### Diabetes knowledge

This study used 8 items to measure diabetes knowledge, including risk factors, diagnosis, symptoms, medical treatments and complications. The items were taken from a prediabetes knowledge questionnaire^31^ providing true and false answer choices. The questionnaire was used with some modifications: the word ‘pre-diabetes’ was replaced with ‘diabetes’ and the phrase ‘fasting plasma glucose values’ was replaced with ‘fasting blood glucose values’ (this was because tests on the latter are more commonly performed). A high score indicated good diabetes knowledge. Cronbach’s alpha was 0.74.

#### EHBM factors

The EHBM contains the factors from the HBM, that is, individual perceptions, life threat, likelihood and cues to action, and the extension includes 2 HBM-modifying factors, that is, LoC and social support.^14,16^

#### Individual perceptions

Individual perceptions included perceived susceptibility and severity which were measured with subscales of diabetes-specific health belief model scales (DSHBs).^32^ Each perceived susceptibility and severity subscale had 2 items. The responses to the 2 items were recorded using a 5-point Likert-type scale from 1 = not severe at all/very unlikely to 5 = extremely severe/very likely. A high score means a high level of perceived susceptibility and severity. Cronbach’s alpha coefficients were 0.92 and 0.73.

#### Modifying factors

Modifying factors included life threat, social support and LoC. Life threat from diabetes was measured by a single item: ‘How much shorter do you think your life expectancy is due to diabetes?’^33^ The response was recorded using a 3-point Likert-type scale from 1 = not at all to 3 = very much shorter. A high score indicates a high life threat.

Social support was measured with the diabetes family behaviour checklist (DFBS) and world value survey (WVS) scale. DFBS contained 16 items, including family support for diet adherence, blood glucose testing, medicines taken, exercise adherence and general support for diabetes care.^34^ The responses were recorded using a 5-point Likert-type scale from 1 = never to 5 = at least once a day. A high score means a high level of family support. Cronbach’s alpha was 0.95.

The WVS scale^35^ contained 17 social capital (SC) items, including bonding SC (trust in group, family, neighbourhood and persons known personally), bridging SC (general trust, trust in persons met for the first time, those from other religions and other nationalities) and linking SC (confidence in the police, the justice system and government). Cronbach’s alpha for each was 0.61, 0.69 and 0.94, respectively. Some of these items are reversely scored. A high total score indicates good SC.

LoC was measured with 3 subscales: internal, external and chance LoC in the DSHBs.^32^ Cronbach’s alpha for each was 0.91, 0.86 and 0.87, respectively. A person with internal LoC considers healthy lifestyle behaviours to be the result of a personal decision, whereas a person with an external LoC considers healthy lifestyle behaviours to be the result of an external factor. On the other hand, a person with chance LoC considers that fate determines healthy lifestyle behaviours. Each subscale had 6 items and the responses were recorded using a 6-point Likert-type scale from 1 = disagree to 6 = agree.

#### Cues to action

Cues to action were measured with a perceived diabetes control subscale from the DSHBs.^32^ The subscale has a single item that reads ‘How well do you think you are managing to control your diabetes?’ The response was recorded using a 5-point Likert-type scale from 1 = not very well to 5 = very well. A high score indicates a higher degree of diabetes control.

#### Likelihood of action

These factors included perceived benefit and barriers, measured with DSHB subscales.^32^ Perceived benefit and barrier subscales have 4 and 5 items, respectively, with responses recorded using a 5-point Likert-type scale from 1 = strongly disagree to 5 = strongly agree. Cronbach’s alphas were 0.93 and 0.84, respectively. High scores indicate great benefits and strong barriers to managing diabetes.

#### Healthy lifestyle behaviours

Healthy lifestyle behaviours were measured with the health lifestyle and personal control questionnaire.^36^ There were 12 items of dietary health choices and dietary harm avoidance, 8 items of daily routine management, 2 items of organized physical exercise and 4 items of social and mental balance. The response was recorded using a 4-point Likert-type scale from 1 = never to 4 = always. Cronbach’s alpha for this questionnaire was 0.95. Cronbach’s alphas for each subscale range from 0.83 to 0.94. A high score indicates healthy lifestyle behaviour.

### Statistical analysis

The data from this study were analysed using SPSS software (version 24.0). Means, percentage and standard deviations (SDs) were determined for descriptive statistics. Pearson’s correlation coefficients were used to identify the correlations among demographic characteristics, clinical and lifestyle factors, diabetes knowledge, EHBM factors and healthy lifestyle behaviour. Predictors of healthy lifestyle behaviour were identified by hierarchical regression analysis. An alpha level of 0.05 was used to determine statistical significance in all statistical tests. Intraclass correlation coefficient (ICC) estimates and their 95% confidence intervals were calculated using SPSS version 24.0 based on a single measurement, absolute-agreement and 2-way mixed-effects model.

### Ethical statement

Hiroshima University ethical committee (reference number E-843) and *Badan* Kesbangpollinmas 8-2016 (Nation unity, Politic and Community Protection Committee) in Buleleng, Bali, Indonesia, approved this study. All procedures in this study followed the ethical standards of the Helsinki Declaration of 1964, as revised in 2013. Written informed consent was obtained from each participant.

## Results

The demographic characteristics, clinical and lifestyle factors, and diabetes knowledge of the 203 participants (Table 1) were as follows. The mean age of the participants was 54.6 years (SD = 8.99). Notably, 36.9% were senior high school graduates, 61.1% took diabetic medicine, mean symptom duration was 33.8 months (SD = 33.3) and 34% consumed alcohol. About 17.7% of the participants believed that diabetes was associated with black magic and shamanism. The mean score for diabetes knowledge was 5.41 (SD = 1.51).

**Table 1.** Demographic characteristics, clinical and lifestyle factors, and diabetes knowledge among patients with type 2 diabetes in rural areas of Bali, August to October 2017 (N = 203).

Variables	Categorization	Mean	SD	Frequency	Percentage
Demographic characteristics					
Age		54.60	8.99		
Sex	Male			115	56.7
	Female			88	43.3
Marital status	Single			5	2.5
	Married			193	95.1
	Divorcee			5	2.5
Education level	No education			19	9.4
	Elementary			41	20.2
	Junior high school			16	7.9
	Senior high school			75	36.9
	Bachelor’s degree			49	24.1
	Graduate			3	1.5
Religion	Hindu			174	85.7
	Moslem			25	12.3
	Buddhist			2	1.0
	Christian			2	1.0
Employment status	Employed			190	93.6
	Retired			6	3.0
	Not employed			7	3.4
Traditional belief–related black magic	Yes			36	17.7
	No			167	82.3
Traditional belief–related shaman	Yes			36	17.7
	No			167	82.3
Clinical and lifestyle factors					
BMI, kg/m^2^	Underweight <18.5			2	1.0
	Normal 18.5-24.9			80	39.4
	Overweight 25-29.9			105	51.7
	Obese ⩾30			16	7.9
Fasting blood glucose level, mg/dL		169.71	62.75		
Symptom duration, mo		33.81	33.32		
	1-12			68	33.5
	13-24			49	24.2
	25-36			21	10.4
	37-48			23	11.3
	49-60			20	9.9
	61-72			7	3.4
	73-84			4	1.9
	⩾85			11	5.4
Another disease	No disease			83	40.9
	Hypertension			76	37.4
	Hyperuricaemia			15	7.4
	Hypertension and hyperuricaemia			12	5.9
	Kidney stones			2	1.0
	Gastritis			3	1.5
	Skin pain			3	1.5
	Vertigo			2	1.0
	Asthma			7	3.4
Diabetic medicine	Yes			124	61.1
	No			79	38.9
Family history of diabetes	Mother			28	13.8
	Father			48	23.6
	No			120	59.1
	Older sibling			4	2.0
	Younger sibling			3	1.5
Tobacco use	Yes			87	42.9
	No			116	57.1
Alcohol use	Yes			69	34.0
	No			134	66.0
Diabetes knowledge (range score: 0-8)		5.41	1.51		

Abbreviation: BMI, body mass index.

The analysis of demographic characteristics and clinical and lifestyle factors (Table 2) revealed that age, education level, religion, employment status, traditional beliefs, fasting blood glucose level, symptom duration, BMI and diabetic medicine were significantly associated with healthy lifestyle behaviours (*P* < .05). In addition, diabetes knowledge (Table 2) was significantly associated with healthy lifestyle behaviours (*P* < .05). The EHBM factors (Table 2) revealed that perceived severity; life threat; family support; bonding SC; internal, external and chance LoC; cues to action; perceived benefit; and barrier were associated with healthy lifestyle behaviours (*P* < .05).

**Table 2.** Pearson’s correlations among independent variables and healthy lifestyle behaviours among patients with type 2 diabetes in rural areas of Bali, August to October 2017 (N = 203).

Variables	Dietary health choices	Dietary harm avoidance	Daily routine	Organized physical exercise	Social and mental balance	Healthy lifestyle behaviour
Demographic characteristics						
Age	–0.232*	–0.115	–0.030	–0.232*	–0.136	–0.162*
Sex	0.047	–0.041	0.091	–0.185*	0.055	0.030
Marital status	0.041	0.009	–0.009	–0.162*	–0.046	–0.021
Education level	0.445*	0.445*	0.379*	0.353*	0.448*	0.506*
Religion	0.144*	0.187*	0.133	0.107	0.052	0.154*
Employment status	–0.248*	–0.218*	0.027	–0.217*	–0.071	–0.148*
Traditional belief–related black magic	0.238*	0.313*	0.414*	0.264*	0.407*	0.413*
Traditional belief–related shaman	0.252*	0.330*	0.424*	0.276*	0.413*	0.427*
Clinical and lifestyle factors						
BMI	–0.123	–0.133	–0.130	–0.121	–0.086	–0.146*
Fasting blood glucose level	–0.395*	–0.324*	–0.213*	–0.184*	–0.340*	–0.359*
Symptom duration	0.149*	0.250*	0.262*	0.041	0.101	0.219*
Other disease	–0.029	–0.036	–0.061	–0.112	–0.101	–0.076
Diabetic medicine	–0.430*	–0.430*	–0.362*	–0.237*	–0.369*	–0.459*
Family history of diabetes	–0.132	–0.070	–0.063	–0.179*	–0.139*	–0.130
Tobacco use	0.022	–0.036	0.149*	–0.215*	0.033	0.037
Alcohol use	–0.093	–0.115	0.099	–0.282*	0.043	–0.037
Diabetes knowledge	0.208*	0.321*	0.448*	0.221*	0.434*	0.419*
Extension of health beliefs						
Perceived susceptibility	0.060	–0.039	–0.082	0.060	–0.016	–0.017
Perceived severity	–0.190*	–0.109	–0.172*	–0.032	–0.034	–0.154*
Life threat	–0.299*	–0.302*	–0.470*	–0.197*	–0.426*	–0.446*
Family support	0.425*	0.514*	0.341*	0.667*	0.431*	0.535*
Bonding SC	0.474*	0.508*	0.250*	0.528*	0.331*	0.473*
Bridging SC	0.022	–0.007	–0.179*	–0.012	–0.128	–0.094
Linking SC	–0.062	0.029	0.126	–0.106	0.191*	0.065
Internal LoC	0.290*	0.407*	0.447*	0.481*	0.351*	0.474*
External LoC	0.180*	0.256*	0.223*	0.333*	0.254*	0.286*
Chance LoC	–0.415*	–0.381*	–0.208*	–0.426*	–0.337*	–0.404*
Cues to action	0.276*	0.412*	0.497*	0.482*	0.466*	0.516*
Perceived benefits	0.297*	0.347*	0.473*	0.324*	0.386*	0.462*
Perceived barriers	–0.363*	–0.424*	–0.586*	–0.351*	–0.481*	–0.564*

Abbreviations: BMI, body mass index: LoC, locus of control; SC, social capital.

* Correlation is significant at .05 level (2-tailed).

Traditional belief in black magic and traditional belief in shamans have a greater variance inflation factor (VIF) than 5 if included in the hierarchical regression equation, in which the VIF value exceeds 5 or 10. It implies that the associated regression coefficients are poorly estimated because of multicollinearity.^37^ It is not recommended to use them in the same regression model as this can cause collinearity and obscure the specific effects of each variable.^38^ Because of that, traditional belief in shamans was not included as a predictor in the hierarchical regression even though they significantly correlated with healthy lifestyle behaviours in the univariate analysis.

The results of the hierarchical regression predicting healthy lifestyle behaviours from demographic characteristics, clinical and lifestyle factors, diabetes knowledge and EHBM are reported in Table 3. All these factors accounted for 71.8% (0.718) of the variance in participants’ healthy lifestyle behaviours. The results of step 1 indicated that the variance accounted for (*R*^2^) with the first 7 predictors from demographic characteristics (age, sex, marital status, education level, religion, employment status and traditional belief–related black magic) equalled 0.339 (adjusted *R*^2^ = 0.315), which was significantly different (*P* < .05). Next, the 8 predictors from clinical and lifestyle factors (symptom duration, BMI, fasting blood glucose level, another disease, diabetes medicine, family history with diabetes, alcohol use and tobacco use) were included in the regression equation. The change in variance accounted for (*R*^2^ change) was equal to 0.126, which shows statistically significant increase in variance accounted for step 1 (*P* < .05). Furthermore, diabetes knowledge was included in the regression equation. The change in variance accounted for (*R*^2^ change) was equal to 0.009, which also shows statistically significant increase in variance accounted for step 2 (*P* < .05). In step 4, EHBM factors (perceived susceptibility; perceived severity; life threat; family support; bonding, bridging and linking SC; internal, external and chance LoC; cues to action; perceived benefit; and perceived barriers) were entered into the regression equation. The change in variance accounted for (*R*^2^ change) was equal to 0.243, which shows a statistically significant increase in variance accounted above the variability contributed by the previous predictor variables entered in step 3 (*P* < .05).

**Table 3.** Hierarchical regression analyses evaluating predictors of healthy lifestyle behaviours among patients with type 2 diabetes in rural areas of Bali, August to October 2017 (N = 203).

Measures	*R* ^2^	Adjusted *R*^2^	*R*^2^ change	Sig. *F* change	Sig. coefficients	Unstandardized coefficients		Standardized coefficients
						*B*	SE	β
Demographic characteristics	0.339	0.315	0.339	<0.001*				
Age					0.003*	0.319	0.104	0.223
Sex					0.131	2.839	1.576	0.92
Marital status					0.616	–1.727	3.442	–0.030
Education level					<0.001*	4.607	0.813	0.480
Religion					0.263	1.845	1.645	0.068
Employment status					0.001*	–0.876	0.252	–0.227
Traditional belief–related black magic					0.007*	6.972	2.551	0.205
Clinical and lifestyle factors	0.465	0.422	0.126	<0.001*				
Symptom duration					0.025*	0.052	0.023	0.136
BMI					0.116	–1.777	1.125	–0.088
Fasting blood glucose level					0.353	–0.013	0.014	–0.062
Another disease					0.396	–0.293	0.344	–0.048
Diabetic medicine					0.002*	–5.638	1.742	–0.204
Family history with diabetes					0.476	–0.654	0.916	–0.042
Alcohol use					0.001*	–7.772	2.397	–0.287
Tobacco use					0.058	4.673	2.451	0.180
Diabetes knowledge	0.474	0.429	0.009	<0.001*				
EHBM factors	0.718	0.670	0.243	<0.001*				
Perceived susceptibility					0.048*	0.821	0.413	0.111
Perceived severity					0.035*	–0.777	0.366	–0.110
Life threat					0.146	–1.700	1.166	–0.083
Family support					<0.001*	0.183	0.049	0.220
Bonding SC					0.017*	0.884	0.368	0.132
Bridging SC					0.808	–0.039	0.160	–0.012
Linking SC					0.297	0.390	0.373	0.053
Internal LoC					0.451	0.131	0.174	0.057
External LoC					0.393	0.123	0.144	0.051
Chance LoC					0.005*	–0.322	0.114	–0.165
Cues to action					0.508	0.584	0.879	0.041
Perceived benefit					0.929	–0.028	0.315	–0.006
Perceived barriers					<0.001*	–0.837	0.197	–0.273

Abbreviations: BMI, body mass index; EHBM, extended health belief model; LoC, locus of control; SC, social capital.

* Correlation is significant at .05 level (2-tailed).

Four of the demographic characteristics (age, education level, employment status and traditional belief–related black magic) were statistically significant (*P* < .05). Next, 3 of clinical and lifestyle factors (symptom duration, diabetes medicine and alcohol use) were statistically significant (*P* < .05). Diabetes knowledge was also statistically significant (*P* < .05). Six of the EHBM factors (perceived susceptibility, severity, family support, bonding SC, chance LoC and perceived barriers) were statistically significant (*P* < .05).

## Discussion

The demographic characteristics, clinical and lifestyle factors, diabetes knowledge and the EHBM proved to be related to participants’ healthy lifestyle behaviours. The demographic characteristic variables were age, education level, employment status and traditional beliefs. The clinical and lifestyle factors were symptom duration, diabetic medicine and alcohol use. The EHBM variables came from the HBM and the extension factors.

The significant variables from demographic characteristics were age, education level and employment status. Age was variable in compliance with lifestyle programmes of patients with type 2 diabetes in studies in Ethiopia and Brazil.^39,40^ The young age patients are associated with the readiness of mentality and motivation to engage in a healthy lifestyle behaviour.^39^ However, a previous literature review of dietary adherence in adults showed opposite result. The result was that adults under 50 years have a higher likelihood of not adhering to a therapeutic diet compared with those aged 50 years and over. Therefore, it is important to consider the age of participants in preparing lifestyle change programmes for patients with type 2 diabetes.

Education level has a positive and significant correlation with healthy lifestyle behaviours. The previous study in the United States^41^ found that low education related strongly to poor health practice in rural area communities. Consequently, efforts to raise awareness of the importance of adopting healthy lifestyle behaviours in patients with type 2 diabetes should be the focus of this group.

Employment status was also a variable for healthy lifestyle behaviour. The work duration in some forms of employment is related to unhealthy dietary practices.^42^ Hence, preparations to overcome the workload in patients with type 2 diabetes can be part of a healthy lifestyle behavioural intervention strategy. In addition, the promotion of healthy diet habits needs to be aimed at young and adult workers.

Traditional beliefs are also a significant variable from demographic characteristics. In Bali, indigenous disease theories are complex and widespread.^43,44^ Metaphysical (black magic) and physical factors are believed by the Balinese to be the 2 main causes of disease.^45^ Therefore, for people in Bali, shamanism has a strong role in curing illnesses and it is often used to complement modern medical care.^46^ This happens throughout Indonesia.^47^ The results of this study showed that traditional beliefs have a significant and positive correlation with healthy lifestyle behaviours, meaning that their practices will be effective in encouraging patients with type 2 diabetes to adopt healthy lifestyle behaviours. Hence, the safety and efficacy of traditional practices should be the main concern of health authorities in Indonesia.

Alcohol use become a significant variable of healthy lifestyle behaviours from clinical and lifestyle factors. This is supported by the previous studies in the United States, Asia and Europe,^48^ which mentioned alcohol intake as a risk factor. Patients with type 2 diabetes who consume alcohol tend to have low adherence to self-care behaviour and are associated with increased morbidity and mortality.^49^ The result of our study showed about 34% of participants consumes the alcohol even though they have diabetes. Drinking is an integral part of indigenous culture in many local communities throughout Indonesia, where it often plays a large role in religious festivals and social gatherings.^50^ Traditionally, in Bali, where the majority religion is Hinduism, alcoholic drink (*arak*) is a symbol of ‘evil’ and is used as a method of calming evil spirits in the human environment.^51^ *Arak* also plays an important role in the secular dimension of Balinese youth culture.^51^ These findings indicate that cultural factor related to alcohol use in Bali could be an important issue that needs to be considered in discussing health-related behaviours. Future work in designing diabetes interventions must be careful and sensitive to this cultural factor and interventions for this group have to be adjusted to this factor.

Symptom duration has also become a significant variable of healthy lifestyle behaviours from clinical and lifestyle factors. The duration of awareness and familiarity with diabetes symptoms can help patients with type 2 diabetes in rural areas of the United States to adopt healthy lifestyle practices.^52,53^ This also occurs in developing countries, as indicated in the findings of this study.

Another significant variable from clinical and lifestyle factors is diabetic medicine. About 61.1% of participants relied on diabetic medicine based on clinical factors data. A previous study in Thailand showed that medication adherence of patients with type 2 diabetes is precisely one of healthy life style practices, which will improve the quality of life.^54^ Hence, strategies for improving diabetes medication adherence are important to be prepared in a diabetes management programme.

Based on clinical and lifestyle factors, the mean age was 54.6 years, 40.9% participants had family history with diabetes and 50.7% had hypertension and hyperuricaemia. These results are in line with the results of clinical studies in rural areas.^55^ Despite the limited technology for the diagnosis and the unavailability of qualified diabetic educators in rural areas,^56^ the health care professionals are expected to recognize risk factors of diabetes in their patients. This is to support early detection and prevention of diabetes, which have to be done as soon as possible in rural area.

High blood glucose level was the cause of another 2.2 million deaths in 2012.^57^ The mean of fasting blood glucose level of the participants was high (169.71 mg/dL). Recent studies have shown that overall good control of blood sugar levels was correlated with decreased incidence of diabetic complications.^58^ This is supported in our study which showed that fasting blood sugar level has negative significant correlations with healthy life style behaviour. It is important to provide comprehensive skills about maintaining blood glucose levels as close as possible to the normal range and practice diabetes management especially in this group to prevent complications.

Diabetes knowledge is a significant variable of healthy lifestyle behaviours. Overall, the participants had moderate scores on diabetes knowledge. They knew that healthy lifestyles could prevent diabetic complications and adopt them to a moderate extent. This is supported by a previous study in Nepal, which stated that patients with type 2 diabetes in health clinics have a much better knowledge about diabetes.^59^ Although diabetes knowledge is a significant factor in supporting healthy lifestyle behaviours in patients with type 2 diabetes, it should not be the main purpose in promoting health.^19,60,61^ This finding is in line with previous research that knowledge acquisition related to diabetes is not enough to improve compliance with diabetes treatment.^25^ Knowledge of certain diseases is 1 component of effective self-management, other components that must be included are behavioural skills, cognitive problem-solving abilities and a sense of efficacy in bringing this ability to influence disease outcomes.^62^ Therefore, the implementation of diabetes knowledge in real behaviour is essential one and needs to be supported with other components.

The previous studies in England and the United States suggested that the EHBM is an adequate and comprehensive model of appraisals of diabetes that affects people’s adherence to diabetes management.^14,16^ This study proved that predictor of healthy lifestyle behaviours was not limited to HBM factors but included extension factors. This means that the EHBM is an important framework for the preparation of diabetes management for rural areas.

Regarding the HBM factors, this study showed that perceived susceptibility and severity were predictors of health behaviours, a view supported by previous studies in Iran and England.^13,14^ In contrast, perceived severity had a negative statistical correlation with healthy lifestyle behaviours. Perceived severity might provoke an emotional response to implement or reject suggested behavioural changes.^63^ This study showed that perceived severity prompts patients with type 2 diabetes to reject behavioural changes. Hence, health care professionals need to assess adults’ emotional responses regarding health beliefs before giving health advice.

The perceived barriers variable from the HBM factors in this study was also a significant predictor of healthy lifestyle behaviours. This is supported by the literature and previous studies in United States and Australia, which stated that perceived barriers are an important predictor of healthy lifestyles.^64-66^ Therefore, health care professionals in rural areas should explicitly explore identification of individual and environmental barriers.

The result of extension factors in the EHBM showed that chance LoC was a significant predictor of healthy lifestyle behaviours. This finding was different from those of previous studies in Germany and Canada that found that all LoC – internal, external and chance – were predictors of healthy behaviour.^67,68^ This result means that fate is believed to determine healthy lifestyle changes for patients with type 2 diabetes in rural Indonesia. However, chance LoC showed an inverse relationship with internal and external LoC, as mentioned in the literature and previous study in Germany.^60,67^ This means that if patients with type 2 diabetes believe in luck or chance, this will increase their unhealthy behaviours because they consider their health as being unrelated to their personal behaviour.

The findings also showed that other extension factors in the EHBM, including family support and bonding SC, were significant associates to healthy lifestyle behaviours. The previous studies both in developed and developing countries indicated that family members^69^ and bonding SC^70-72^ were the closest social support for patients with type 2 diabetes and were expected to play important roles in supporting their healthy lifestyle behaviours. In addition, family influences are particularly strong for patients with type 2 diabetes in rural areas, so adults who receive family support tend to practice healthy lifestyle behaviours. Therefore, family members and those in the immediate environment of patients with type 2 diabetes should be involved in diabetes management for this area.

Few studies have investigated demographic characteristics, clinical and lifestyle factors, diabetes knowledge and the EHBM extensively in adults with type 2 diabetes in rural Indonesia. Thus, this study can be as initial data in designing diabetes prevention and management in rural areas.

This study also has several limitations such as diversity of participants and generalization of the study because it was undertaken in the rural areas in Bali. Further research needs to involve a more diverse participant sample. This would be useful to explore the healthy lifestyles of patients with type 2 diabetes in other contexts. The significant negative correlation between diabetic medicine and healthy lifestyle practices also needs to be investigated further. Furthermore, the clinical and lifestyle factors were obtained by self-report which might be limited because of its recall bias. The researchers already assessed both of demographic characteristics and clinical factors^73^ and used regression analysis to adjust for confounders,^74^ but there might be other confounders that have not be measured. Finally, this study was cross-sectional and could make no inferences regarding cause and effect concerning healthy lifestyle behaviour among adults with type 2 diabetes in rural Indonesia.

## Conclusions

The findings from this study indicate that demographic characteristics, clinical and lifestyle factors, diabetes knowledge and the EHBM could predict healthy lifestyle behaviours in patients with type 2 diabetes in the rural areas of Bali. The extension factors of EHBM, that is, family support, bonding SC and chance LoC, are an adequate framework that could help to predict healthy lifestyles among patients with type 2 diabetes in rural areas. Therefore, efforts to promote healthy behaviours by patients with type 2 diabetes should not be limited to educating them about diabetes but should also consider their health beliefs, support from the family and those in the environment, and culture around them. The following recommendations are offered. At the individual patient level, the EHBM can be used in diabetes education classes including important information about the cause and complications of diabetes, improving diabetes knowledge and perceived susceptibility, also reducing risk factors and the benefits of behaviour change. At the level of family, friends and small groups, the diabetes class can intentionally encourage participants to bring family members and/or friends to class and facilitate the development of a friendly atmosphere in the classroom itself. The aim is to make participants feel supported, both interpersonally and in a real way, throughout the class. Such efforts would be more effective in increasing the healthy lifestyle practice and reducing the risk of diabetes complications.
